# Linkage of blood cell division cycle 42 with T helper cells, and their correlation with anxiety, depression, and cognitive impairment in stroke patients

**DOI:** 10.1590/1414-431X2023e12855

**Published:** 2023-09-08

**Authors:** Haifeng Ma, Qing Chang, Jujuan Jia, Yaoyuan Zhang, Gang Wang, Yuanyuan Li

**Affiliations:** 1 Department of Neurology, The First Affiliated Hospital of Hebei North University, Zhangjiakou, China

**Keywords:** Stroke, Cell division cycle 42, T helper cells, Anxiety and depression, Cognitive impairment

## Abstract

Cell division cycle 42 (CDC42) regulates T helper (Th) cell differentiation and is related to psychological disorders. This study aimed to assess the correlation between blood CDC42 and Th cells, and their association with mental issues in stroke patients. Peripheral blood samples were obtained from 264 stroke patients and 50 controls. Then, serum CDC42 was measured by enzyme-linked immunosorbent assay, and Th1, Th2, and Th17 cells were detected by flow cytometry. Hospital Anxiety and Depression Scale (HADS) and Mini Mental State Examination (MMSE) were applied to patients. CDC42 was decreased (P<0.001), Th1 (P=0.013) and Th17 (P<0.001) cells were elevated, while Th2 cells (P=0.108) showed no difference in stroke patients compared to controls. In addition, CDC42 was negatively associated to Th1 (P=0.013) and Th17 (P<0.001) cells in stroke patients but were not associated with Th2 cells (P=0.223). Interestingly, CDC42 was negatively associated with HADS-anxiety (P<0.001) and HADS-depression scores (P=0.034) and positively associated with MMSE score (P<0.001) in stroke patients. Lower CDC42 was associated to lower occurrence of anxiety (P=0.002), depression (P=0.001), and cognitive impairment (P=0.036) in stroke patients. Furthermore, increased Th17 cells were positively correlated with HADS-anxiety and HADS-depression scores and inversely correlated with MMSE score, which were also associated with higher occurrence of anxiety, depression, and cognitive impairment in stroke patients (all P<0.05). Blood CDC42 and Th17 cells were correlated, and both of them were linked to the risk of anxiety, depression, and cognitive impairment. However, the findings need further large-scale validation, and the implicated mechanism needs more investigation.

## Introduction

Stroke is a cerebral blood circulation disorder that may further induce neurological deficit, which is one of the main causes of disability and death in the global population (1,2). In addition, post-stroke mental disorders including anxiety, depression, and cognitive impairment have become an important clinical concern (1,3,4), negatively affecting the quality of life and disease rehabilitation in stroke patients (5,6). Through the deep understanding of the pathogenesis of these mental disorders, the role of immune dysfunctions driven by cluster of differentiation 4^+^ (CD4^+^) T cells in promoting the development of mental and cognitive problems has been recognized gradually (7-9). For example, one previous study shows that mitochondrial fission in mice CD4^+^ T cells causes anxiety, depression, and other behavioral abnormalities (10). Another study suggests that the increase of T helper (Th) 17 cells promotes cognitive impairment in mice (9). Therefore, the study of CD4^+^ T cells and its regulators is of great significance in improving the mental health and cognitive function of stroke patients, which may further benefit their clinical prognosis.

Cell division cycle 42 (CDC42), as one of the members of the Rho-guanosine triphosphatase family, regulates the differentiation of CD4^+^ T cells, which may have a potential role in mental health and cognitive function (11,12). Moreover, CDC42 is also reported to participate in mental disorders and cognitive impairment (13,14). One study indicates that inhibition of CDC42 increases anxiety in mice (13). Another study reports that overexpression of CDC42 in mice can ameliorate the cognitive impairment caused by isoflurane (14). Based on the above research, it is reasonable to hypothesize that CDC42 and CD4^+^ T cells are involved in the pathogenesis of psychological and cognitive disorders and are relevant to these symptoms in stroke patients. Therefore, this study aimed to evaluate the relationship between CDC42 and CD4^+^ T cells, as well as their correlations with anxiety, depression, and cognitive impairment in stroke patients.

## Material and Methods

### Participants

In this study, a total of 264 first-admission or re-admission stroke patients were consecutively enrolled from September 2020 to April 2022. The inclusion criteria were: 1) diagnosed as ischemic stroke per Chinese guidelines for diagnosis and treatment of acute ischemic stroke 2018 (15); 2) age greater than 18 years; 3) Mini Mental State Examination (MMSE) score >20 and capable of understanding the study protocol and complete the corresponding scale assessment; and 4) cooperate with the collection of peripheral blood (PB) samples. The exclusion criteria were: 1) use of immunosuppressive agents that affect the circulating immune system currently; 2) had immune system disease or active infection; and 3) lactation or pregnant women. During the same period, fifty controls were enrolled. The inclusion criteria were: 1) age- and sex-matched with stroke patients; and 2) had at least two stroke risk factors, e.g., hypertension, atrial fibrillation, diabetes mellitus, dyslipidemia, smoking, overweight, etc (16). The exclusion criteria were: 1) a history of stroke or subclinical stroke; 2) had taken immunosuppressive agents that affect the circulating immune system currently; 3) immune system disease or active infection; and 4) pregnancy or lactation. This research had approval from the Ethics Committee of The First Affiliated Hospital of Hebei North University (China), and each subject or his/her guardian provided written informed consent.

### Data and sample collection

Clinical characteristics of stroke patients, including demographics, comorbidities, and disease-related information, were collected after enrollment. MMSE score at enrollment was also collected. PB samples of all subjects were also obtained after enrollment and stored at 4°C for a short period of time before detection. Serum was isolated from half of the PB samples and stored at -80°C, while the other half of the PB samples were used immediately for the detection of Th1, Th2, and Th17 cells.

### Sample analysis

The levels of CDC42 in serum were measured by commercial enzyme-linked immunosorbent assay (ELISA) kits (No. Cat. YJ908876, Shanghai Enzyme-linked Biotechnology, China). The CD4^+^ T cells were isolated from PB samples using a Dynabeads™ FlowComp™ Human CD4 kit (No. Cat. 11361D, Thermo Fisher Scientific, USA). Then, the levels of Th1, Th2, and Th17 cells in CD4^+^ T cells were measured by flow cytometry analysis, using commercial human Th1/Th2/Th17 phenotyping kits (No. Cat. 560751, BD, USA). All tests were performed in strict accordance with the kit instructions.

### Evaluation

For stroke patients, the Hospital Anxiety and Depression Scale for anxiety (HADS-A) and HADS for depression (HADS-D) scores and MMSE score were evaluated before discharge. The total score of each sub-scale is 21 points (HADS-A >7 indicates anxiety; HADS-D >7 indicates depression) (17). The MMSE was performed to assess cognitive impairment, and the highest score was 30 (>27 points indicates cognitive impairment) (18).

### Statistical analysis

SPSS v.22.0 (IBM Corp., USA) and GraphPad Prism v.7.01 (GraphPad Software Inc., USA) were used for data analysis and figure plotting, respectively. The Wilcoxon rank sum test was used to compare the difference between groups. The Spearman test was used for correlation analysis. The receiver-operating characteristic (ROC) was employed to demonstrate the capacity of CDC42 to discriminate stroke patients from controls. Backward stepwise-multivariate logistics regression models were used for finding factors related to anxiety, depression, and cognitive impairment among stroke patients. P<0.05 was considered to be statistically significant.

## Results

### Baseline features of stroke patients

A total of 264 stroke patients with a mean age of 67.4±9.5 years were enrolled, including 173 (65.5%) males and 91 (34.5%) females. There were 72 (27.3%) stroke patients who had recurrences. Moreover, the mean values of the HADS-A, HADS-D, and MMSE scores were 7.2±2.6, 7.5±2.7, and 27.5±1.5, respectively. In all stroke patients, there were 83 (31.4%) patients with anxiety, 100 (37.9%) patients with depression, and 47 (17.8%) patients with cognitive impairment. More detailed characteristics of stroke patients are shown in Table 1.

**Table 1:** Characteristics of stroke patients.

Characteristics	Stroke patients (n=264)
Age (years), mean±SD	67.4±9.5
Gender, n (%)	
Male	173 (65.5)
Female	91 (34.5)
BMI (kg/m^2^), mean±SD	24.0±2.6
Education, n (%)	
Primary school or below	62 (23.5)
Junior high school	78 (29.5)
High school	78 (29.5)
University or above	46 (17.5)
Marriage status, n (%)	
Married	103 (39.0)
Single/divorced/widowed	161 (61.0)
Location, n (%)	
Urban	47 (17.8)
Rural	217 (82.2)
History of smoking, n (%)	135 (51.1)
Hypertension, n (%)	226 (85.6)
Hyperlipidemia, n (%)	119 (45.1)
Diabetes mellitus, n (%)	63 (23.9)
CKD, n (%)	49 (18.6)
CVD, n (%)	91 (34.5)
Primary lesion site, n (%)	
Left	125 (47.3)
Right	87 (33.0)
Bilateral/brainstem/unknown	52 (19.7)
Recurrence experience, n (%)	72 (27.3)
HADS-A score, mean±SD	7.2±2.6
Anxiety, n (%)	83 (31.4)
HADS-D score, mean±SD	7.5±2.7
Depression, n (%)	100 (37.9)
MMSE score, mean±SD	27.5±1.5
Cognitive impairment, n (%)	47 (17.8)

SD: standard deviation; BMI: body mass index; CKD: chronic kidney disease; CVD: cardiovascular disease; HADS-A: Hospital Anxiety and Depression Scale for Anxiety; HADS-D: Hospital Anxiety and Depression Scale for Depression; MMSE: Mini Mental State Examination.

### Comparison of CDC42, Th1, Th2, and Th17 between stroke patients and controls

**Figure 1:**
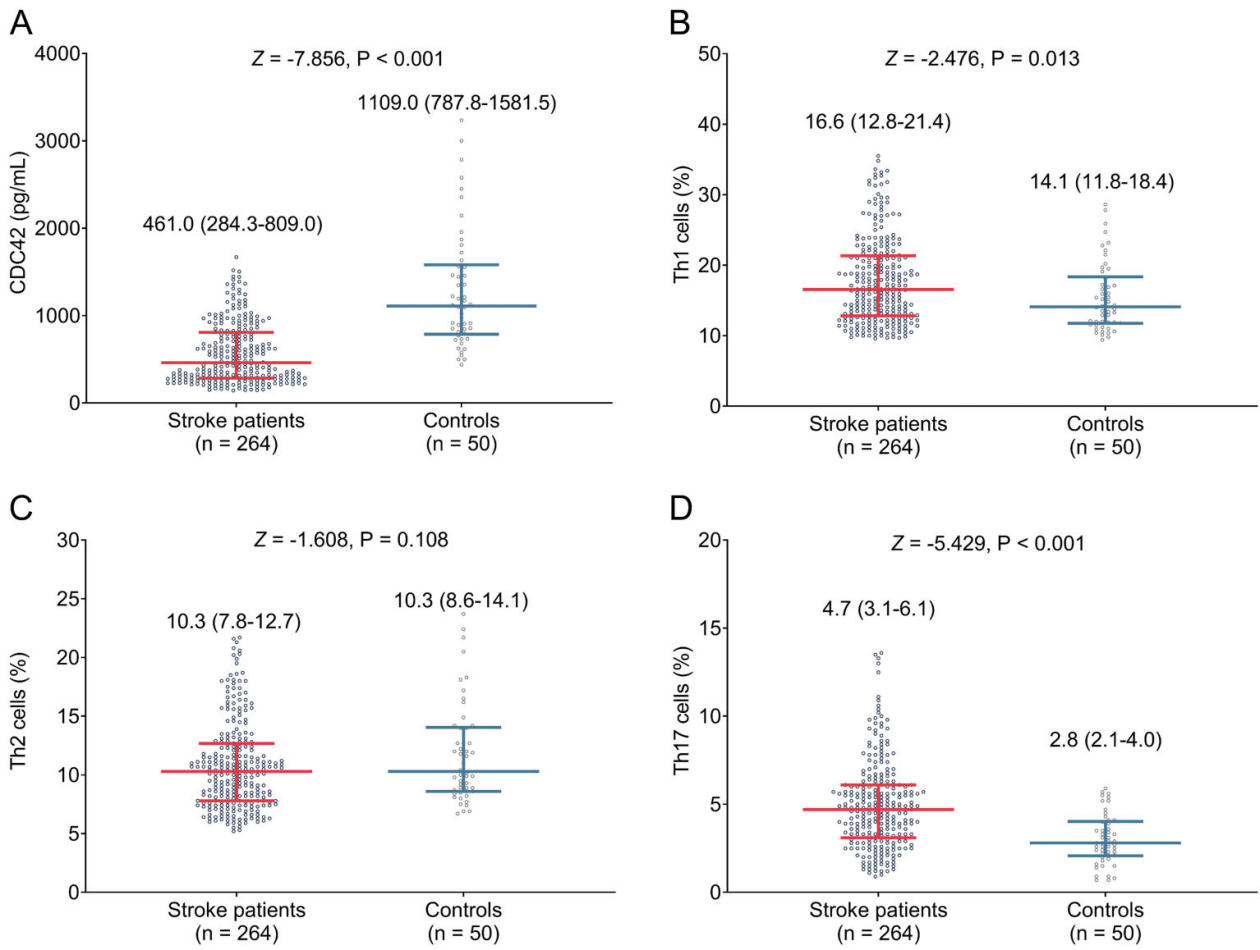
Cell division cycle 42 (CDC42) (**A**), Th1 cells (**B**), Th2 cells (**C**), and Th17 cells (**D**) of stroke patients and controls. Data are reported as median and interquartile range (Wilcoxon rank sum test).

CDC42 was reduced in stroke patients compared with controls (P<0.001) (Figure 1A). However, Th1 cells were increased in stroke patients compared to controls (P=0.013) (Figure 1B). No difference was found in Th2 cells between groups (P=0.108) (Figure 1C). Th17 cells were elevated in stroke patients compared with controls (P<0.001) (Figure 1D). Furthermore, CDC42 showed a good value to distinguish stroke patients from controls with area under curve of 0.850 (95% confidence interval: 0.801-0.900) (Supplementary Figure S1).

### Correlation of CDC42 with Th1, Th2, and Th17 in stroke patients

**Figure 2:**
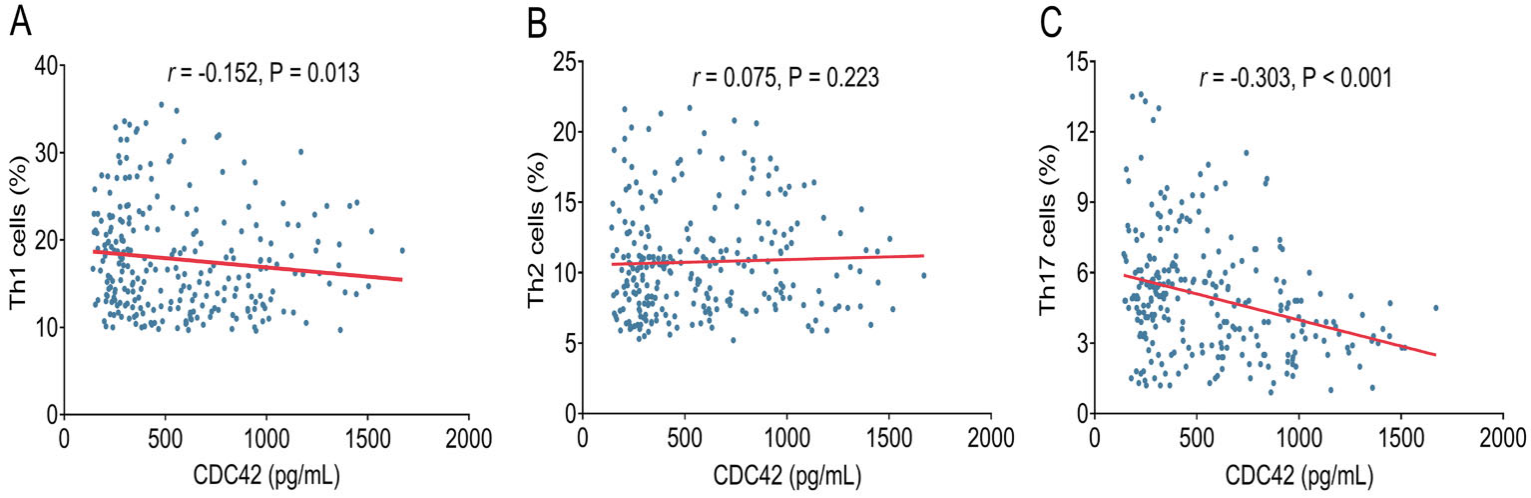
Correlation of cell division cycle 42 (CDC42) with Th1 cells (**A**), Th2 cells (**B**), and Th17 cells (**C**) in stroke patients.

CDC42 was inversely associated with Th1 cells (r=-0.152, P=0.013) (Figure 2A), while there was no relationship between CDC42 and Th2 cells (*r*=0.075, P=0.223) (Figure 2B). Furthermore, CDC42 was negatively correlated to Th17 cells (r=-0.303, P<0.001) (Figure 2C) in stroke patients.

### Correlation of CDC42, Th1, Th2, and Th17 with anxiety in stroke patients

**Figure 3:**
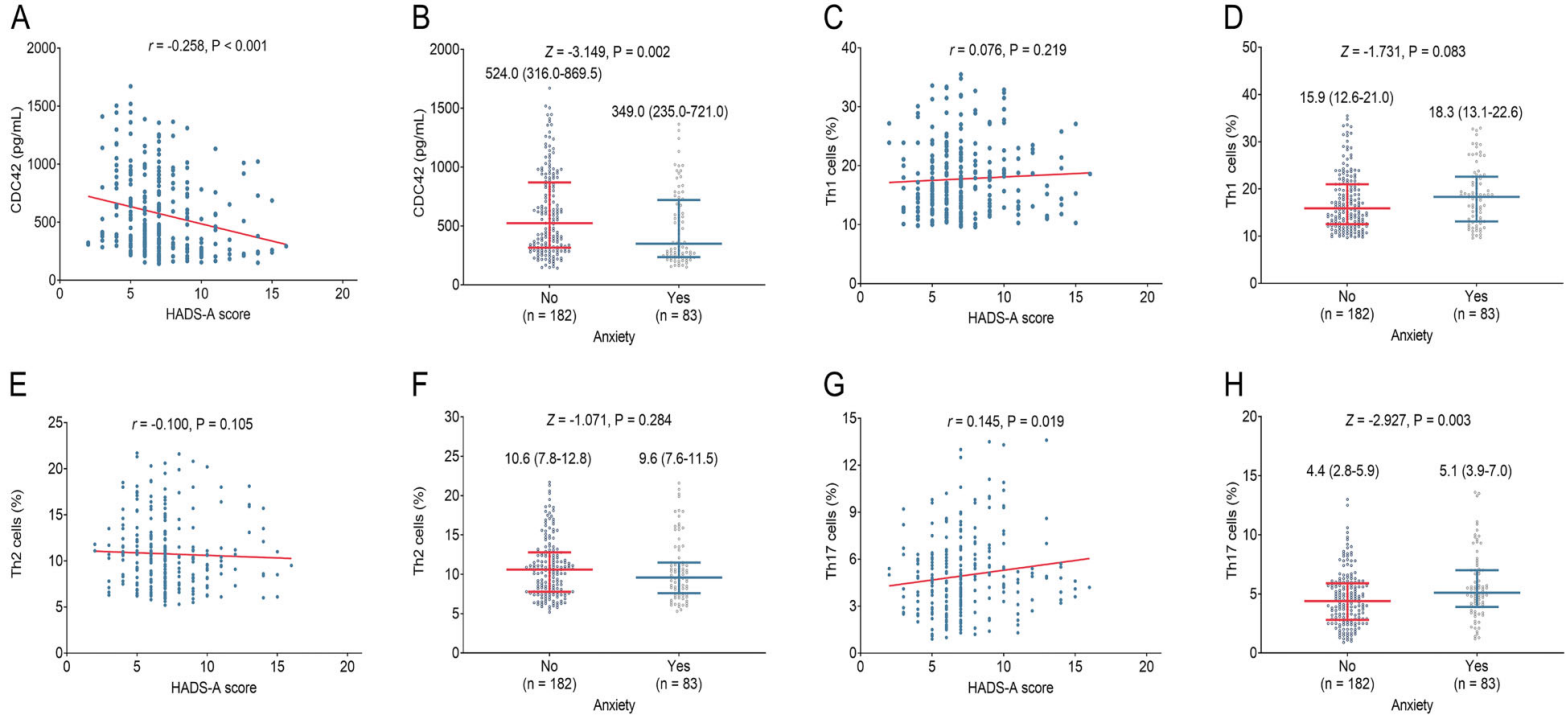
Relationship between cell division cycle 42 (CDC42) and Hospital Anxiety and Depression Scale (HADS)-A (anxiety) score (**A**) and anxiety (**B**); between Th1 cells with HADS-A score (**C**) and anxiety (**D**); between Th2 cells with HADS-A score (**E**) and anxiety (**F**); and between Th17 cells with HADS-A score (**G**) and anxiety (**H**) in stroke patients. **A**, **C**, **E**, and **G**, Spearman correlation. **B**, **D**, **F**, and **H**: Data are reported as median and interquartile range (Wilcoxon rank sum test).

CDC42 was negatively correlated with HADS-A score (r=-0.258, P<0.001) (Figure 3A), and low CDC42 was correlated with the occurrence of anxiety in stroke patients (P=0.002) (Figure 3B). Th1 cells were not associated with HADS-A score (r=0.076, P=0.219) (Figure 3C) or anxiety (P=0.083) (Figure 3D) in stroke patients. Similarly, there was no correlation of Th2 cells with HADS-A score (r=-0.100, P=0.105) (Figure 3E) or anxiety (P=0.284) (Figure 3F) in stroke patients. However, Th17 cells were positively correlated with HADS-A score (r=0.145, P=0.019) (Figure 3G), and were associated with the occurrence of anxiety in stroke patients (P=0.003) (Figure 3H).

### Correlation of CDC42, Th1, Th2, and Th17 with depression in stroke patients

**Figure 4:**
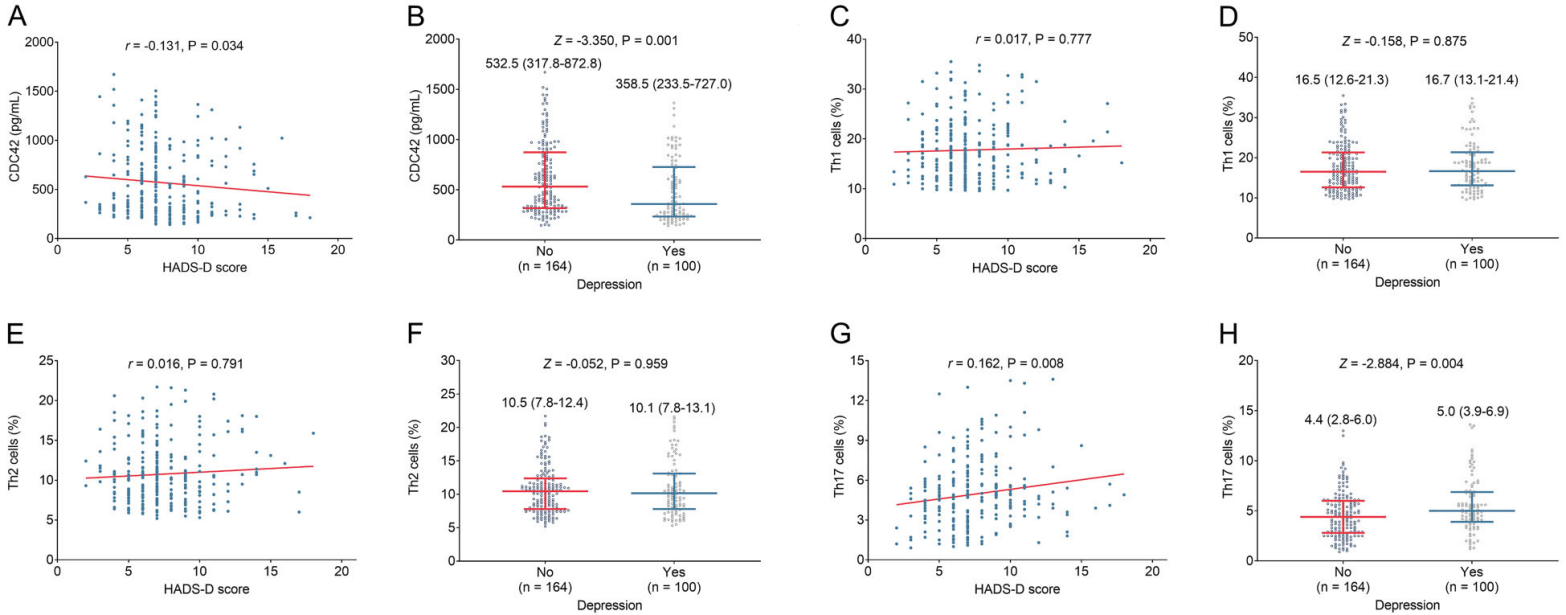
Relationship between cell division cycle 42 (CDC42) and Hospital Anxiety and Depression Scale (HADS)-D (depression) score (**A**) and depression (**B**); between Th1 cells and HADS-D score (**C**) and depression (**D**); between Th2 cells and HADS-D score (**E**) and depression (**F**); and between Th17 cells and HADS-D score (**G**) and depression (**H**) in stroke patients. **A**, **C**, **E**, and **G**, Spearman correlation. **B**, **D**, **F**, and **H**: Data are reported as median and interquartile range (Wilcoxon rank sum test).

CDC42 was inversely correlated with HADS-D score (r=-0.131, P=0.034) (Figure 4A), and low CDC42 was correlated with the occurrence of depression in stroke patients (P=0.001) (Figure 4B). However, Th1 cells were not related to HADS-D score (r=0.017, P=0.777) (Figure 4C) or depression (P=0.875) (Figure 4D) in stroke patients. There was no relationship of Th2 cells with HADS-D score (r=0.016, P=0.791) (Figure 4E) or depression (P=0.959) (Figure 4F) in stroke patients. Notably, Th17 cells were positively correlated with HADS-D score (r=0.162, P=0.008) (Figure 4G) and with the occurrence of depression in stroke patients (P=0.004) (Figure 4H).

### Correlation of CDC42, Th1, Th2, and Th17 with cognitive impairment in stroke patients

**Figure 5:**
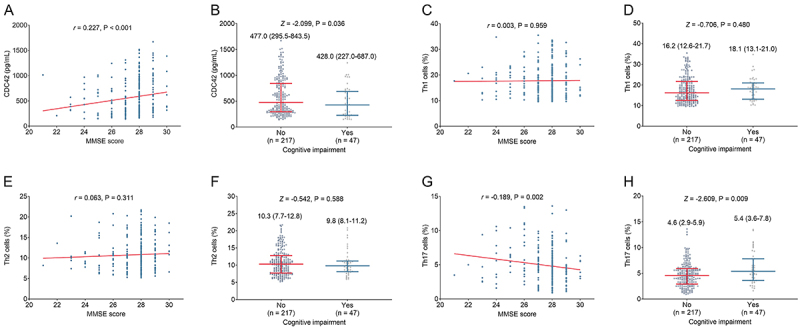
Relationship between cell division cycle 42 (CDC42) and Mini Mental State Examination (MMSE) score (**A**) and cognitive impairment (**B**); between Th1 cells and MMSE score (**C**) and cognitive impairment (**D**); between Th2 cells and MMSE score (**E**) and cognitive impairment (**F**); and between Th17 cells and MMSE score (**G**) and cognitive impairment (**H**) in stroke patients. **A**, **C**, **E**, and **G**, Spearman correlation. **B**, **D**, **F**, and **H**: Data are reported as median and interquartile range (Wilcoxon rank sum test).

CDC42 was positively associated with MMSE score (r=0.227, P<0.001) (Figure 5A), and low CDC42 was correlated to the occurrence of cognitive impairment in stroke patients (P=0.036) (Figure 5B). However, Th1 cells were not correlated with MMSE score (r=0.003, P=0.959) (Figure 5C) or cognitive impairment (P=0.480) (Figure 5D) in stroke patients. In addition, there was no correlation of Th2 cells with MMSE score (r=0.063, P=0.311) (Figure 5E) or cognitive impairment (P=0.588) (Figure 5F) in stroke patients. Furthermore, Th17 cells were negatively correlated to MMSE score (r=-0.189, P=0.002) (Figure 5G) and with the occurrence of cognitive impairment in stroke patients (P=0.009) (Figure 5H).

Interestingly, the multivariate logistics regression models revealed that higher CDC42 was independently associated with lower risk of anxiety (odds ratio=0.401, P=0.043), but not with depression or cognitive impairment in stroke patients (Supplementary Table S1).

### Subgroup analysis according to recurrence experience in stroke patients

In stroke patients without recurrence experience, low CDC42 was associated with the occurrence of anxiety (P=0.038) and depression (P=0.004), while it was not related to the occurrence of cognitive impairment (P=0.293). The percent of Th17 cells was correlated with the occurrence of anxiety (P=0.035) and depression (P=0.045), but it was not correlated with the occurrence of cognitive impairment (P=0.069). Additionally, Th1 cells and Th2 cells were not correlated with anxiety, depression, or cognitive impairment in those patients (all P>0.05) (Table 2).

**Table 2:** Subgroup analysis based on recurrence experience in stroke patients.

Items	CDC42 (pg/mL)median (IQR)	Th1 cells (%)median (IQR)	Th2 cells (%)median (IQR)	Th17 cells (%)median (IQR)
Without recurrence experience				
Anxiety				
No	481.0 (314.0-911.0)	16.3 (13.2-21.4)	10.5 (7.8-12.7)	4.5 (2.8-5.9)
Yes	380.0 (245.5-732.0)	18.2 (13.3-22.9)	9.9 (8.3-12.6)	5.1 (3.8-7.0)
P value	0.038	0.465	0.959	0.035
Depression				
No	528.0 (319.5-918.0)	16.7 (13.6-21.8)	10.1 (7.7-12.1)	4.6 (2.8-6.0)
Yes	380.0 (235.0-721.0)	16.6 (13.1-21.7)	10.4 (8.3-13.1)	4.9 (3.8-6.8)
P value	0.004	0.642	0.297	0.045
Cognitive impairment				
No	461.0 (294.3-902.8)	16.5 (13.1-21.7)	10.3 (7.8-12.8)	4.7 (2.8-6.0)
Yes	484.0 (241.3-703.5)	17.9 (13.6-22.5)	9.8 (8.2-11.4)	4.9 (3.6-7.7)
P value	0.293	0.530	0.933	0.069
With recurrence experience				
Anxiety				
No	602.5 (322.3-806.8)	13.9 (11.3-18.8)	10.9 (8.2-13.1)	4.1 (2.9-6.3)
Yes	272.5 (227.0-627.0)	18.8 (13.0-22.3)	9.1 (7.0-11.2)	5.3 (4.0-8.2)
P value	0.011	0.022	0.074	0.019
Depression				
No	601.0 (312.0-792.0)	14.5 (11.6-19.8)	10.9 (8.2-12.4)	4.0 (2.9-6.3)
Yes	349.0 (229.5-778.0)	17.9 (12.3-21.3)	9.1 (6.9-13.7)	5.1 (4.0-7.9)
P value	0.088	0.237	0.132	0.016
Cognitive impairment				
No	601.0 (298.5-813.5)	15.2 (11.7-21.5)	10.5 (7.6-13.3)	4.5 (3.3-5.5)
Yes	312.0 (227.0-593.0)	18.8 (12.6-19.8)	9.8 (7.6-11.1)	5.6 (3.6-9.3)
P value	0.039	0.335	0.447	0.053

CDC42: cell division control protein 42; Th1: helper 1; Th2: T helper 2; Th17: T helper 17.

In stroke patients with recurrence experience, low CDC42 was correlated with the occurrence of anxiety (P=0.011) and cognitive impairment (P=0.039), while it was not correlated with the occurrence of depression (P=0.088). Furthermore, the percent of Th1 cells was associated with the occurrence of anxiety (P=0.022), but it was not associated with the occurrence of depression (P=0.237) or cognitive impairment (P=0.335). Subsequently, the percent of Th17 cells was correlated with the occurrence of anxiety (P=0.019) and depression (P=0.016), while it was not correlated with the occurrence of cognitive impairment in those patients (P=0.053). Additionally, the percent of Th2 cells was not associated with anxiety, depression, or cognitive impairment (all P>0.05) (Table 2).

## Discussion

CDC42 is thought to be involved in the adjustment of the immune response by regulating CD4^+^ T cells (19,20). For instance, a previous study suggests that CDC42 inhibits the differentiation of Th17 cells in mice (20). Another study reported that CDC42 aggravated the Th1/Th2 cell imbalance and inhibited Th17 cell differentiation (19). Regarding clinical practices, a study found that CDC42 is positively correlated with Th2 cells and inversely correlated with Th17 cells in Alzheimer's disease patients (21). Another study also found a positive association of CDC42 with Th2 cells and a negative association of CDC42 with Th17 cells in acute ischemic stroke patients (12). Our study found that CDC42 was inversely correlated with Th1 cells and Th17 cells but not associated with Th2 cells in stroke patients. The possible explanations were that either CDC42 inhibited Th1 cell differentiation by activating the p21 protein-activated kinase 1 signal pathway and inhibiting extracellular signal-regulated kinase-mediated T-cell receptor signaling (22) or that CDC42 inhibited Th17 cell differentiation by inhibiting glycolysis (20).

One study reported that inhibition of brain CDC42 activity causes anxiety in mice (13). Nevertheless, the relationship between CDC42 and mental health in stroke patients has not been reported. In our study, low CDC42 was associated with the occurrence of anxiety and depression to some extent in stroke patients. This might be due to 1) CDC42 knockdown could mediate a trafficking/functionality defect in γ-aminobutyric acid type B receptors, resulting in anxiety and depression (23); or 2) CDC42 knockdown might promote the synthesis of xanthine by regulating the differentiation of CD4^+^ T cells, thus leading to anxiety, depression, and other behaviors (10). Furthermore, the involvement of CDC42 in cognitive impairment is also noteworthy. A previous report found that overexpression of CDC42 alleviates cognitive impairment in mice (13). Another recent study reports that CDC42 is positively correlated with the MMSE score in Alzheimer's disease patients (21). Similarly, our study revealed that CDC42 was positively associated with MMSE score, and low CDC42 was related to the occurrence of cognitive impairment in stroke patients. This might be because 1) The deficiency of CDC42 affected the synaptic plasticity in hippocampal neurons, resulting in the decline of cognitive ability (24); or 2) The deficiency of CDC42 induced blood-brain barrier interruption and neuroinflammation by regulating Th cell differentiation, which further caused cognitive impairment (25).

In addition to CDC42, the association of CD4^+^ T cells with mental health and cognitive function is also a key issue in stroke patients' management. A previous study shows that Th17 cells are correlated with an increased risk of anxiety, depression, and cognitive impairment in patients with gastric cancer (26). Another study observed that Th17 cells are correlated with an increased rate of cognitive impairment in acute ischemic stroke patients (27). Our study showed that Th17 cells were correlated to the occurrence of anxiety, depression, and cognitive impairment, but Th1 and Th2 cells were not linked with the above mental and cognitive problems in stroke patients. The possible reasons were as follows: 1) Th17 cells secreted interleukin-17A, which enhanced the release of neurotransmitters in the medial prefrontal cortex, leading to brain injury by inducing the structural remodeling of microglia and promoting a continuous inflammatory reaction, thus causing anxiety and depression (28,29); or 2) Th17 cells might regulate cognitive function by regulating hippocampal neurogenesis, neuroinflammation, etc. (30,31).

There were some limitations in this study: 1) Although the sample size was relatively large, further studies should consider including an even greater sample size to obtain a clearer conclusion; 2) Our study only used the HADS to assess stroke patients' anxiety and depression, and further studies should consider using multiple assessment scales for investigation; 3) The mismatch of the number of stroke patients and controls might interfere with the statistical effect.

In conclusion, CDC42 was negatively correlated with Th17 cells, and both were associated with psychiatric disorders in stroke patients, indicating that their monitoring may contribute to the management of mental and cognitive problems after stroke.

## Supplementary Material

Click here to view [pdf].
